# Experimental Demonstration of 3 × 3 MIMO LED-to-LED Communication Using RGB Colors

**DOI:** 10.3390/s21144921

**Published:** 2021-07-20

**Authors:** Hyunwoo Jung, Sung-Man Kim

**Affiliations:** Department of Electronic Engineering, Kyungsung University, Nam-gu, Busan 48434, Korea; hyunwoo125@hotmail.com

**Keywords:** LED-to-LED, Li-Fi, light-emitting diodes, optical wireless communication, visible light communication

## Abstract

Optical wireless communication (OWC) is one of the promising candidates for beyond fifth-generation communication (B5G). Depending on the type of transmitters, receivers, and information carriers applied in the system, OWC can be categorized into visible light communication, light fidelity, free-space optical communication, optical camera communication, etc. In addition to these OWC subcategories, this paper proposes light-emitting diode (LED)-to-LED communication as another subcategory of OWC technique. Furthermore, we show an experimental demonstration of the multiple-input multiple-output (MIMO) LED-to-LED communication system using red, green, and blue colored LEDs. We believe that LED-to-LED communication is an effective solution to resolve the communication burden arising from massive connectivity in B5G internet of things. Along with the measurement results of the transmitter LED, receiver LED, and the channel properties, it is shown that the MIMO LED-to-LED system is able to successfully recover the transmitted signal with low inter-channel interferences due to the receiver LED’s unique characteristics. Finally, the bit error rate (BER) performance of the MIMO LED-to-LED system is shown in comparison with the BER performance of the single-input single-output (SISO) LED-to-LED system. We successfully implemented the 3 × 3 MIMO LED-to-LED communication system using RGB colors at a data rate of 30.62 kbps over a 10 cm transmission distance along with direct current biased optical orthogonal frequency division multiplexing (DCO-OFDM) modulation and zero-forcing (ZF) equalizer.

## 1. Introduction

Since the advent of the first-generation (1G) wireless mobile communication, demand for a high-speed communication system has been increasing exponentially. In response to this demand, the fifth-generation (5G) communication is currently being deployed to provide lower latency, higher data rate, higher security, massive connectivity, and ubiquitous wireless communication services. One of the major differences between 5G and the previous generations of the communication systems is that the 5G communication system exploits a millimeter-wave (mm-wave) as an information carrier because the current 3 kHz to 30 GHz radiofrequency (RF) band does not have enough bandwidth to support complete 5G communication services. As an extension of this trend, one can expect that the mm-wave band will be exhausted in the near future and a higher frequency spectrum will be required.

Optical wireless communication (OWC) is one of the very promising candidates for future indoor wireless communication that transmits information via nanometer-waves such as infrared (IR), visible light (VL), and ultraviolet (UV). Typically, the OWC system uses light-emitting diodes (LEDs) or laser diodes (LDs) for transmitters, and photodiodes (PDs) or image sensors (ISs) for receivers. OWC has several advantages compared to its RF counterpart. Firstly, OWC has an enormous bandwidth. The near-IR to near-UV waveband ranges from 214.3 to 1000 THz, which is approximately 2500 times larger than the RF (3 kHz–300 GHz) region. Secondly, the infrastructure for the OWC system is already well-prepared. Since OWC uses LEDs for its transmitter, LEDs placed in buildings, traffic lights, or streetlights can be employed as optical signal transmitters. Thus, OWC can realize ubiquitous wireless communication at low cost. Thirdly, optical waves cannot pass through opaque objects. Therefore, the optical signal can be easily confined indoors, ensuring high security and high-frequency/wavelength reuse communication. Due to its advantages, extensive research has been performed on OWC. In [1], the authors outline the requirements of the 5G, B5G, and internet of things (IoT) techniques, and show that OWC can meet these requirements. 

**Figure 1:**
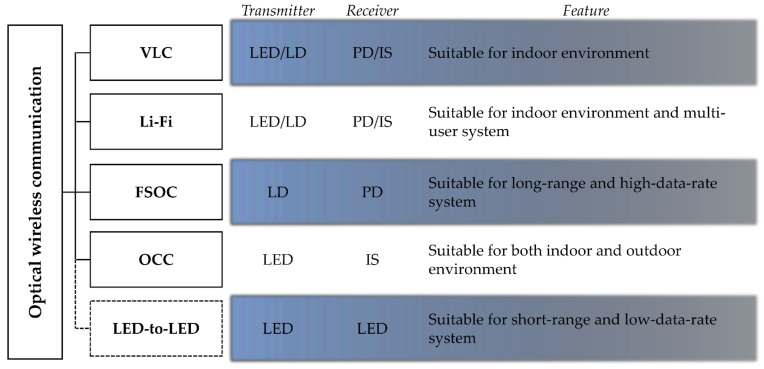
Subcategories of optical wireless communication.

Depending on the type of transmitter and receiver, communication distance, and exploited waveband in the system, OWC can be subcategorized into visible light communication (VLC), light-fidelity (Li-Fi), free-space optical communication (FSOC), optical camera communication (OCC), etc. [2]. Each of the OWC sub-techniques are shown in Figure 1. Typically, VLC uses the VL spectrum for the downlink (DL) and uplink (UL) communication, LED/LD for its transmitter, and PD/IS (photodiode or image sensor) for its receiver. For high-speed communication, the multiple-input multiple-output (MIMO) technique can be applied to the VLC system. However, the realization of the MIMO VLC system is quite challenging due to highly correlated channel conditions [3,4,5]. Similar to VLC, Li-Fi uses LED/LD for its transmitter and PD/IS for its receiver. The MIMO technique is applicable in the Li-Fi system, yet challenging due to the highly correlated channel condition [6]. The difference between Li-Fi and VLC is that (i) Li-Fi uses the VL spectrum for the DL and IR/VL/UV for the UL, on the other hand, VLC uses the VL spectrum for both DL and UL, and (ii) VLC includes both point-to-point (P2P) and point-to-multipoint (P2MP) communication, while Li-Fi includes only P2MP communication. FSOC uses LD and PD for the transmitter and receiver and transmits data via the IR/VL/UV spectrum. The FSOC communication scheme aims only for P2P communication. MIMO is applicable to the FSOC system as atmospheric turbulence and scintillation by long-distance transmission ensure a low correlation of each channel [7]. OCC uses the IR/VL waveband and LED and IS for its transmitter and receiver. Since optical cameras are composed of millions of pixels, optical signals from different directions can be separated into images [8]. Therefore, OCC is considered one of the most ideal candidates for optical MIMO. Further information on each OWC subcategory is well-described in [9,10,11,12].

The LED-to-LED technique is a communication scheme employing LEDs for both transmitters and receivers, and transmits the information via the IR/VL/UV spectrum. Our previous work shows that LEDs can be utilized not only as optical transmitters but also as optical receivers because the basic physical structures of LEDs and PDs are the same. The LED-to-LED communication system has an interesting characteristic whereby receiver LEDs can generate photocurrents only when they receive light with shorter wavelengths than the wavelength they were designed to as a transmitter. This property allows LED-to-LED communication to form a unique channel matrix, ensuring sufficiently low correlated channels. Thereby, unlike indoor VLC or Li-Fi, the MIMO scheme is applicable in LED-to-LED communication.

In wireless communications, the importance of ultra-dense networks (UDN) and IoT have been increasing to support higher network capacity and massive connectivity services. However, there are difficulties in realizing UDN and IoT. Since small cells such as picocells and femtocells need to be deployed, realizing UDN is expected to have high costs. The implementation of IoT will induce tremendous data traffic increases as a large number of end-user smart devices, cars, industrial utilities, and sensors will be connected to each other, consuming vast frequency bandwidth even for simple control signal transmission.

We believe that LED-to-LED communication can be a complementary solution for a UDN or IoT application. As LED-to-LED communication schemes can be easily setup by using already-deployed LEDs in the indoors, streets, vehicles, and smart devices, UDN can be realized without further investment to develop densely located infrastructures. Furthermore, the use of vast IR/VL wavebands can relieve the communication burden of sending billions of simple control signals generated by massive device connections. We are also expecting that LED-to-LED communication can be used as a step for the OWC commercialization strategies.

To our knowledge, the studies on LED-to-LED communication are not comprehensively conducted. The interchangeability between solid-state light emission and detection was widely known by Forest W. Mims in the 1970s, and a subsequent study on LED-to-LED communication began in 2003 [13,14]. In [15], the characterization of single-color power LEDs as photodetectors was experimentally measured and a fair comparison between LEDs and silicon a photodetector with respect to their spectral, temporal, and spatial properties was made. In [16], a LED-to-LED ad-hoc network was suggested together with a software-based physical layer and medium access control layer for sensor networks, smart and connected consumer devices, and IoT. In [17], the performance dependency of the LED-to-LED communication system depending on the color of the transmitter and receiver LEDs was studied. In [18], the optimization method choosing the colors of the transmitter and receiver LEDs was shown to maximize the channel capacity according to Shannon’s channel capacity law. However, the LED-to-LED-based MIMO techniques are not studied yet.

Therefore, in this paper, we propose a LED-to-LED communication system as another subcategory of OWC and experimentally demonstrate 3 × 3 MIMO LED-to-LED communication using RGB colors. In the following sections, after consideration of various MIMO and modulation schemes, we show the mathematical model of our system. In Section 3, the characteristics of the transmitter LEDs, receiver LEDs, and LED-to-LED channels are measured. Furthermore, we compare the bit error rate (BER) performance of the MIMO LED-to-LED communication system and single-output (SISO) LED-to-LED communication. Finally, we summarize the paper with a conclusion.

## 2. Digital Signal Processing Model of the 3 × 3 MIMO LED-to-LED System

Orthogonal frequency division multiplexing (OFDM) modulation ensures strong resistance to inter-symbol interference (ISI) and high spectral efficiency by simultaneously transmitting data via orthogonally located subcarriers. Therefore, the OFDM modulation scheme is often employed in a wireless communication system to improve channel capacity. On the other hand, MIMO simultaneously transmits data via different propagation paths to achieve great spectral efficiency and high data rates. In [19], the performance comparison of spatial multiplexing (SMP), repetition coding (RC), and spatial modulation (SM) MIMO algorithms for indoor OWC was studied. When sufficiently low channel correlation and signal-to-noise ratio (SNR) are guaranteed, SMP achieved better BER performance and higher spectral efficiency compared to RC and SM. Thereby, MIMO together with OFDM modulation are widely used in wireless communication systems to maximize the spectral efficiency. The LED-to-LED communication system has a limited channel capacity and noise vulnerability due to the low responsivity of the receiver LED. To overcome such disadvantages, we apply the OFDM modulation together with the SMP MIMO algorithm to our 3 × 3 LED-to-LED communication scheme.

Since OFDM for RF communication cannot be applied to intensity modulation and direct detection (IM/DD)-based OWC systems, several optical OFDM techniques, such as direct current biased optical OFDM (DCO-OFDM), asymmetric clipped optical OFDM (ACO-OFDM), and asymmetrically clipped direct current biased OFDM (ADO-OFDM), were developed [20]. In our system, DCO-OFDM is applied since it is simple and has a high spectral efficiency compared to ACO-OFDM and ADO-OFDM.

**Figure 2:**
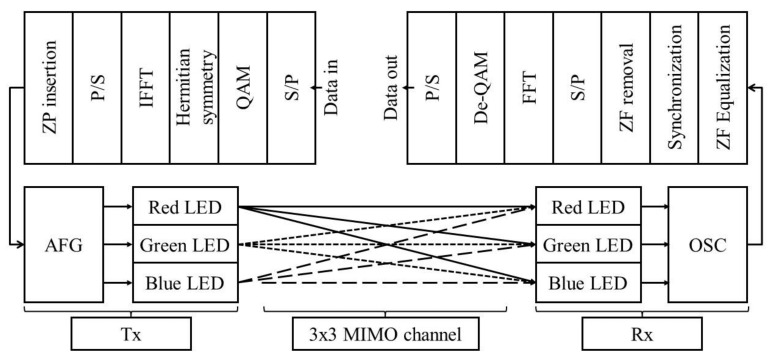
Block diagram of the 3 × 3 MIMO LED-to-LED communication system.

Figure 2 shows the block diagram of the 3 × 3 MIMO LED-to-LED communication system using RGB LEDs. Information bitstreams for each red, green, and blue transmitter are converted from serial-to-parallel (S/P) and mapped onto the complex-valued symbols according to quadrature amplitude modulation (QAM). Generated QAM symbols are then extended to have Hermitian symmetry for real-valued OFDM signals. The resulting frequency-domain symbols for the *q*th transmitter, $d_{q}=\left[\;d_{q,0}\;d_{q,1}\ldots \;d_{q,N-1}\;\right]$, meet the following conditions:

(1) $$\left\{\begin{array}{c} d_{q,0}=d_{q,\frac{N}{2}-1}=0\\ d_{q,k}=d_{q,N-k}^{*} \end{array}\right.,\;\;k=1,\ldots ,\frac{N}{2}-1$$

Afterwards, frequency-domain symbols are transformed into the discrete *n*th time-domain OFDM signal, $x_{q,n}$, by the inverse fast Fourier transform (IFFT) process:

(2) $$x_{q,n}=\frac{1}{\sqrt{N}}\overset{N-1}{\underset{k=0}{\sum }}d_{q,k}\exp\left(\frac{j2\pi nk}{N}\right)=\frac{1}{\sqrt{N}}\overset{\frac{N}{2}-1}{\underset{k=0}{\sum }}\left\{d_{q,k}\exp\left(\frac{j2\pi nk}{N}\right)+d_{q,k}^{*}\exp\left(-\frac{j2\pi nk}{N}\right)\right\}$$

where *N* is the size of fast Fourier transform (FFT), *j* is the imaginary unit, and ${\left(.\right)}^{*}$ is the conjugate operator. After parallel-to-serial (P/S) conversion and zero padding (ZP) insertion, the resulting signals are fed into an arbitrary function generator (AFG) to drive each red, green, and blue LED with direct current (DC) bias. The optical power at the *n*th time can be provided as:

(3) $$P_{q,n}=\left(x_{q,n}+DC_{q}\right)\cdot C_{E/O,q}$$

where $DC_{q}$ denotes DC bias at the *q*th transmitter, and $C_{E/O,q}$ is electrical current-to-optical power (E/O) conversion gain at the *q*th transmitter.

Assuming transmitter LEDs are Lambertian radiators, the generalized luminous intensity can be expressed as:

(4) $$I\left(\phi_{q}\right)=\frac{m+1}{2\pi }cos^{m}\left(\phi_{q}\right)$$

where $m=-\frac{\ln2}{\ln\left(\cos\phi_{q,1/2}\right)}$ is the order of Lambertian emission, and $\phi_{q}$ is the *q*th LED’s angle of irradiance. The emitted light signals then propagate through the free space channel to the receiver LEDs. The free space channel impulse response from the *q*th transmitter LED to the *p*th receiver LED is provided as:

(5) $$h_{p,q}=\left[\;h_{p,q,0}\;h_{p,q,1}\ldots \;h_{p,q,L-1}\;\right]$$

where *L* denotes the length of the channel impulse response. In indoor OWC, the gain of non-line-of-sight (NLOS) components is relatively low compared to the gain of the line-of-sight (LOS) component, and the channel frequency response is flat from DC to 3 dB channel bandwidth [21]. Thereby, the channel impulse response can be approximated as LOS DC channel gain:

(6) $$h_{p,q}\approx h_{p,q,0}=h_{p,q}^{DC}$$

The DC channel gain can be calculated as follows:

(7) $$h_{p,q}^{DC}=\left\{\begin{array}{c} \frac{A_{p}}{d_{p,q}^{2}}I\left(\phi_{q}\right)\cos\left(\varphi_{p,q}\right),\;\;0\leq \varphi_{p,q}\leq \varphi_{p,c}\\ 0,\;\;\varphi_{p,q}>\varphi_{p,c} \end{array}\right.$$

where $\varphi_{p,c}$ is the field of view (FOV) of the *p*th received LED, and $A_{p}$ is the *p*th receiver LED’s collection area, as follows:

(8) $$A_{p}=\frac{a^{2}A_{LED,p}}{\sin\varphi_{p,c}}$$

where *a* is the concentrator refractive index of the *p*th LED, and $A_{LED,p}$ is the *p*th LED area. The received *n*th time domain signal at the *p*th receiver LED can be represented as:

(9) $$r_{p,n}=\overset{N_{t}}{\underset{q=1}{\sum }}P_{q,n}\cdot h_{p,q}^{DC}\cdot R_{p,q}+w_{p,n}=\overset{N_{t}}{\underset{q=1}{\sum }}\left(x_{q,n}+DC_{q}\right)\cdot C_{E/E,p,q}+w_{p,n}$$

where $R_{p,q}$ denotes responsivity of the *p*th receiver at the *q*th transmitter LED’s wavelength, $P_{q,n}$ is the optical power of the *q*th transmitter LED at the *n*th OFDM symbol, $w_{p,n}$ is the mean square noise current, and $C_{E/E,p,q}$ is the electrical current-to-electrical current (E/E) conversion gain. Finally, the *n*th transmitted OFDM symbol can be recovered by the zero-forcing (ZF) equalizer:

(10) $${\hat{x}}_{n}=H^{-1}r_{n}$$

The rest of the demodulation process can be performed in the reverse order of the modulation process. 

## 3. MIMO LED-to-LED Communication Using RGB Colors

**Figure 3:**
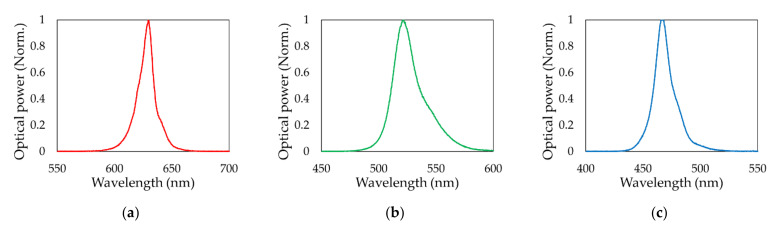
Emission spectra of the (**a**) red, (**b**) green, and (**c**) blue LEDs.

**Figure 4:**
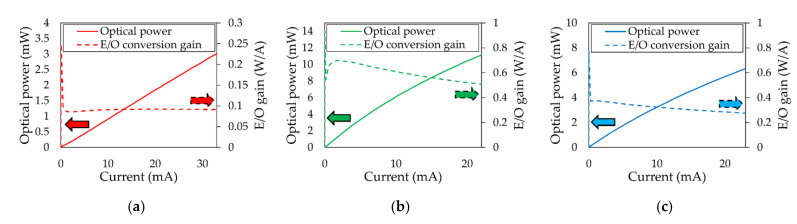
Optical power and E/O efficiency of the (**a**) red, (**b**) green, and (**c**) blue LEDs with respect to the input current.

All experiments conducted in this paper used off-the-shelf red, green, and blue LEDs. The diameter of the LED is 10 mm, and the emission angle is 30 degrees. Note that the emission angle was measured with respect to the LED’s light emission center line, at which the radiant intensity falls to half of its maximum value. Figure 3 shows the wavelength spectrum of the (a) red, (b) green, and (c) blue LEDs. The peak wavelengths of red, green, and blue LEDs were measured at 629, 521, and 466 nm, respectively. Figure 4 shows the optical power and E/O conversion efficiency of the (a) red, (b) green, and (c) blue LEDs with respect to input current.

**Figure 5:**
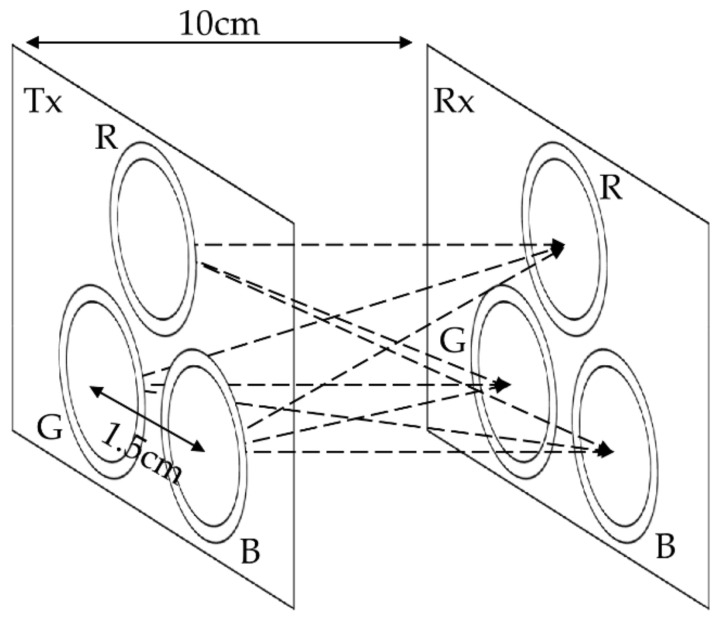
The geometry of the 3 × 3 MIMO LED-to-LED communication system.

Figure 5 illustrates the geometry of the MIMO LED-to-LED communication system. Transmitter LEDs were located 1.5 cm apart from each other. The distance between the transmitter plane and the receiver plane was set to 10 cm. All MIMO experiments were performed under the geometry condition shown in Figure 5.

**Figure 6:**
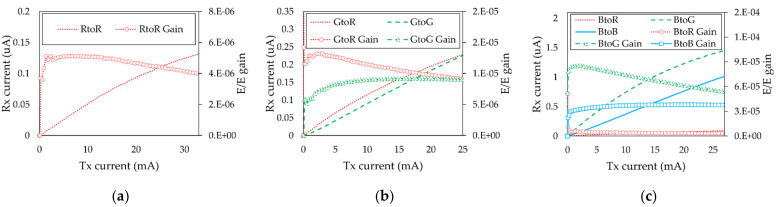
Output current and channel DC gain with respect to the input current when transmitter color is (**a**) red, (**b**) green, and (**c**) blue. The reason why RtoG, RtoB, and GtoB cases are not displayed is that the receiver LEDs cannot receive longer wavelength signals than the wavelength they were designed to as a transmitter.

The responsivity and frequency response of the optical receiver varies depending on the applied wavelength of the light. Therefore, we measured the channel DC gain and frequency response in all possible color combinations to observe the channel characteristics of the LED-to-LED system. Figure 6 shows the output current and E/E conversion efficiency at the red, green, and blue receivers with respect to the transmitter input current when the transmitter LED color is (a) red, (b) green, and (c) blue. The results show that the receiver LED cannot generate optical current when its wavelength is shorter than the transmitter wavelength. Thus, the MIMO LED-to-LED communication system forms a unique channel condition, as follows:

(11) $$\left[\begin{array}{c} r_{r,n}\\ r_{g,n}\\ r_{b,n} \end{array}\right]=\left[\begin{array}{ccc} h_{r,r}^{DC} & h_{r,g}^{DC} & h_{r,b}^{DC}\\ h_{g,r}^{DC} & h_{g,g}^{DC} & h_{g,b}^{DC}\\ h_{b,r}^{DC} & h_{b,g}^{DC} & h_{b,b}^{DC} \end{array}\right]\left[\begin{array}{c} x_{r,n}\\ x_{g,n}\\ x_{b,n} \end{array}\right]=\left[\begin{array}{ccc} h_{r,r}^{DC} & h_{r,g}^{DC} & h_{r,b}^{DC}\\ 0 & h_{g,g}^{DC} & h_{g,b}^{DC}\\ 0 & 0 & h_{b,b}^{DC} \end{array}\right]\left[\begin{array}{c} x_{r,n}\\ x_{g,n}\\ x_{b,n} \end{array}\right]$$

By solving Equation (11), the transmitted signal can be recovered ensuring sufficiently low channel correlation between each channel. 

ZF-based MIMO systems are known to cause noise amplification effects at receivers [22]. Therefore, different amounts of power were applied to red, green, and blue transmitter LEDs to ensure sufficient levels of SNR. The blue-to-blue channel had no channel interference from the green and red transmitter LEDs, which resulted in a minimum signal power allocation to the blue transmitter LED. Since the green-to-green channel was interfered by a blue transmission signal, a higher signal power than the blue signal was applied to the green transmitter LED. Finally, the red-to-red channel was interfered by green and blue signals. Thus, we allotted the highest power to the red transmitter LED. The rest of the experiments were carried out by applying 0.94 to 2.75 mA for the blue LED, 9.4 to 25 mA for the green LED, and 0.27 to 36.4 mA for the red LED.

**Figure 7:**
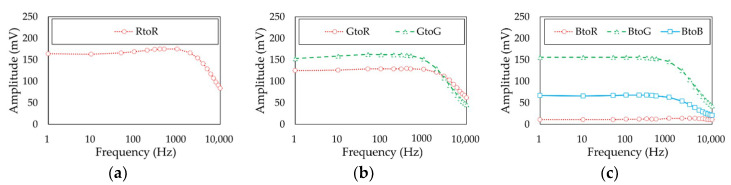
Frequency response of the channel when the transmitter is (**a**) red, (**b**) green, and (**c**) blue.

Under such current allocations onto each color of the LED, Figure 7 shows the frequency response results when the transmitter LED is (a) red, (b) green, and (c) blue. The red-to-red, green-to-green, and blue-to-blue channel 3 dB bandwidths were measured as 6, 3, and 3 kHz, respectively.

**Figure 8:**
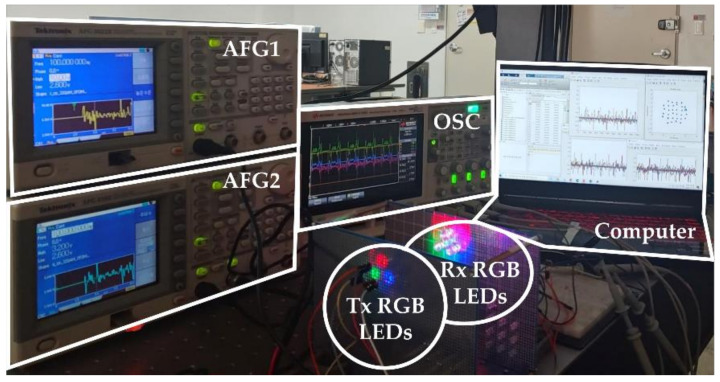
Experimental setup of the 3 × 3 MIMO LED-to-LED communication system using RGB LEDs.

Figure 8 illustrates the experimental setup of a 3 × 3 MIMO LED-to-LED communication system using RGB colors, where 160-bit pseudo-random binary sequence (PRBS) bit streams for each channel are mapped to a QAM symbol. The complex-valued QAM symbols were modified by adding a redundancy satisfying Hermitian symmetry, as shown in Equation (1). Afterward, IFFT processing was performed to load data symbols onto orthogonally located subcarriers. The resulting signals appear as a real value. After adding ZPs and DC bias, the OFDM signals were directly modulated with arbitrary function generators (AFG) to drive each red, green, and blue LED. After a 10 cm transmission, emitted visible light signals were detected by a red, green, and blue receiver LED and recorded by an oscilloscope (OSC). The received signals were recovered in the sequence of ZF equalization, FFT, and QAM de-mapping. Digital signal processing was carried out via MATLAB. Detailed experimental parameters are listed in Table 1. 

We measured the BER performance of the 3 × 3 MIMO LED-to-LED system by varying the bit-rate, and compared the results with the SISO LED-to-LED systems. Note that the bit-rate of each LED channel in the MIMO LED-to-LED system was set to be equal.

**Figure 9:**
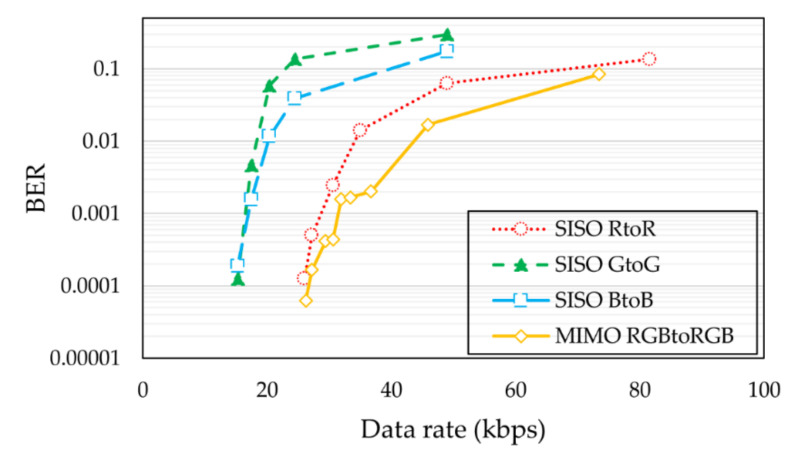
BER of the SISO LED-to-LED systems and 3 × 3 MIMO LED-to-LED system.

The BER performances of the 3 × 3 MIMO LED-to-LED communication system and the SISO LED-to-LED communication systems are shown in Figure 9. Note that the SISO LED-to-LED system uses the same geographical and power allocation as the MIMO LED-to-LED system. Assuming that a BER of 10^−3^ is a communication-capable threshold, the maximum data rates of the SISO red-to-red, SISO green-to-green, SISO blue-to-blue, and MIMO RGB-to-RGB were measured at 27.2, 15.31, 15.31, and 30.62 kbps, respectively.

**Figure 10:**
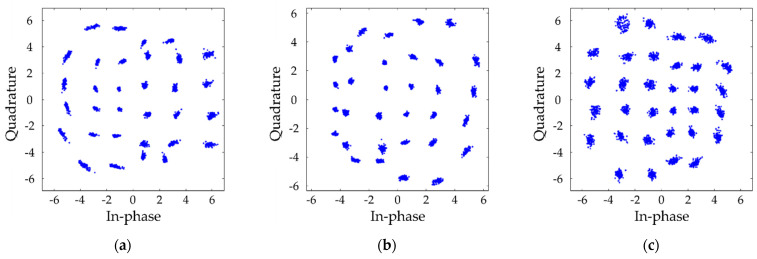
Constellation diagram of SISO LED-to-LED system. (**a**) RtoR, (**b**) GtoG, and (**c**) BtoB when transfer data rate is 27.2, 15.31, and 15.31 kbps, respectively.

**Figure 11:**
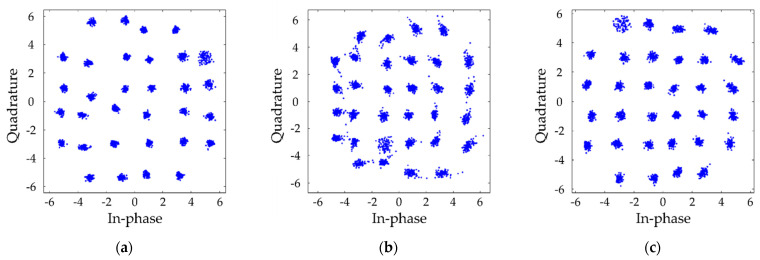
Constellation diagram of MIMO LED-to-LED system. (**a**) RtoR, (**b**) GtoG, and (**c**) BtoB when transfer data rate of each channel is 10.21 kbps.

Figure 10 shows the constellation diagram of (a) RtoR, (b) GtoG, and (c) BtoB SISO LED-to-LED system when the transfer data rate of each channel is 27.2, 15.31, and 15.31 kbps, respectively. Figure 11 shows the constellation diagram of each (a) RtoR, (b) GtoG, and (c) BtoB channel of the MIMO LED-to-LED system when the transfer data rate of each channel is equally set to be 10.21 kbps. Table 2 shows the BER performance of the 3 × 3 MIMO LED-to-LED communication system at a transmission distance of 10 and 15 cm. Note that all the experimental settings except the transmit distance were the same. Our results show that the communications are almost impossible at 15 cm. The results show that the BER performance of the system varies significantly in terms of the transmission distance. In the future work, we need to find a way to increase the transmission distance.

In [23], performance analysis of a MIMO VLC system using different equalizers was studied. The ZF equalizer, zero-forcing with successive interference cancellation (ZF-SIC) equalizer, and minimum mean squared error with successive interference cancellation (MMSE-SIC) equalizer were tested in 2 × 2 and 4 × 4 MIMO conditions. The results were good in the order of MMSE-SIC, ZF-SIC, and ZF equalizers. Thereby, we think that the MIMO LED-to-LED system could be further improved with the ZF-SIC or MMSE-SIC equalizer.

In this work, we have experimentally demonstrated the feasibility of the LED-to-LED MIMO OWC system using off-the-shelf LEDs. The results show that MIMO LED-to-LED communication could successfully recover the transmitted signal with low inter-channel interferences using the receiver LED’s wavelength-selective characteristics. Obviously, the data rate of the LED-to-LED communication system is low compared to other OWC subcategories. However, the advantage of LED-to-LED communication is that the LED-to-LED communication system is able to realize low-data IoT at the lowest costs in comparison with other OWC schemes. We believe that LED-to-LED communication is an effective solution to resolve the communication burden arising from massive connectivity in B5G IoT. Additional research issues such as communication distance, modulation, equalization, etc., will be handled in our future work.

## 4. Conclusions

In this paper, we proposed LED-to-LED communication as another subset of OWC, because LED-to-LED communication is not only an effective solution for UDN and IoT but can also be a commercialization strategy for OWC. Secondly, we presented mathematical models and experimental demonstrations of 3 × 3 MIMO LED-to-LED communication systems using RGB colors. We have shown that MIMO LED-to-LED communication with RGB LEDs forms sufficiently low correlated channels due to the special characteristics of the LED as an optical receiver, for which the acceptable wavelength range varies depending on its color. The MIMO LED-to-LED communication experiments were conducted with a QAM DCO-OFDM modulated signal, the SMP MIMO algorithm, and the ZF equalizer. Assuming a communicable threshold of a 10^−3^ BER, the maximum reachable data rate of the MIMO LED-to-LED communication was 30.62 kbps at a 10 cm transmission distance. Additionally, the results showed that the MIMO LED-to-LED system outperforms SISO LED-to-LED systems. We expect that the system can be improved further by applying a ZF-SIC or MMSE-SIC equalizers in the MIMO LED-to-LED system.

## Figures and Tables

**Table 1:** Experimental conditions in 3 × 3 MIMO LED-to-LED communication system.

Parameter		Value	Parameter		Value
LED diameter		10 mm	QAM level		32
LED angle of emission		30 deg.	OFDM subcarrier number		66
Transmission distance		10 cm	Zero padding length		34
Peak wavelength	R	629 nm	Operating current	R	0.27–36.3 mA
	G	521 nm		G	9.4–25 mA
	B	466 nm		B	0.94–2.75 mA
Average E/O gain	R	0.0909	Average E/E gain	RtoR	$4.62\times 10^{-6}$
	G	0.56		GtoR	$1.02\times 10^{-5}$
	B	0.3715		GtoG	$9.12\times 10^{-6}$
System bandwidth	R	6000 Hz		BtoR	$5.49\times 10^{-6}$
	G	3000 Hz		BtoG	$8.52\times 10^{-5}$
	B	3000 Hz		BtoB	$3.2\times 10^{-5}$

**Table 2:** BER comparison of the 3 × 3 MIMO LED-to-LED system at a transmission distance of 10 and 15 cm.

		BER Results at a Transmission Distance of	
Data Rate (kbps)		10 cm	15 cm
8.17	RtoR	$<6.25\times 10^{-5}$	0.025
	GtoG	$<6.25\times 10^{-5}$	0.019
	BtoB	$6.25\times 10^{-5}$	$5.63\times 10^{-4}$
8.17×3	RGBtoRGB	$2.08\times 10^{-5}$	0.015
9.8	RtoR	$<6.25\times 10^{-5}$	0.055
	GtoG	$1.25\times 10^{-3}$	0.081
	BtoB	$<6.25\times 10^{-5}$	$6.25\times 10^{-4}$
9.8×3	RGBtoRGB	$4.17\times 10^{-4}$	0.046
12.25	RtoR	$2.93\times 10^{-3}$	0.098
	GtoG	$3.12\times 10^{-3}$	0.11
	BtoB	$<6.25\times 10^{-5}$	$2.69\times 10^{-3}$
12.25×3	RGBtoRGB	$2.02\times 10^{-3}$	0.071

## Data Availability

Not applicable.
